# Personal and Book Notices

**Published:** 1876-11

**Authors:** 


WE have been pleased to welcome to our city and State Dr. Greensville Dowell, Professor of Surgery in the Medical College of Texas.

Dr. Dowell has, as many of our readers are aware, invented a method for the radical cure of hernia—both in the construction of instruments and the manner of operating. He has embodied his discovery, with many interesting cases, in a good size volume which will soon be given to the profession. He has operated over seventy times without a single death, and with very few failures. This is a good showing.

The doctor is also the author of a work on yellow fever, which will be noticed hereafter in our pages.

It may be well to say that Dr. Dowell has performed his operation for the cure of hernia in Virginia and Georgia within the last month—with what results the future will determine. We wish him abundant success, as he has all the enthusiasm, nerve, energy and talent of the able surgeon. G.

DR. JOHN A. OCTERLONY has resigned his chair in the Louisville Medical College and Kentucky School of Medicine. His course in this particular will meet the approval of the profession, and puts him right on the record.

*Scribner's Illustrated Monthly.*—Dr. Holland, the editor of Scribner—one of the most beautiful and chaste writers in our country—will commence a new serial story in the December number, to be entitled "Nicholas Minturn." From a knowledge of the previous merits of Dr. Holland's stories, we know the readers of Scribner have a delightful treat in store. Dr. Holland is an M. D.—one of us: a good and worthy brother of the medical fraternity.

*St. Nicholas,* the best and freshest magazine for the young—and *old*—in the world, will have a new serial story in the December number. Let all the boys read it. Let all the fathers subscribe for it. Published by Scribner & Co., New York. Price, $3.00.

THE *Popular Science Monthly* is the scientific journal of the continent. It is always brim-full of scientific matter, from the pens of the ablest men of Europe and America. All questions of science are discussed in its pages by men fully up to the times—discussed by such men as Tyndall, Spencer, Carpenter and others.

Published by D. Appleton & Co., New York. Price, $5.00.

